# Wellness in Every Stage of Life: A New Paradigm for Public Health Programs

**Published:** 2006-12-15

**Authors:** Karen Steinberg

**Affiliations:** Centers for Disease Control and Prevention, Coordinating Center for Health Promotion

In 1992, Congress changed the name of CDC from the Centers for Disease Control to the Centers for Disease Control *and Prevention* (1) to reflect the widening of CDC's mission, which increasingly had come to include the actual prevention of disease as well as its control. Today, the concept of "wellness" is being introduced into CDC's public health lexicon as CDC strives to meet its overarching health protection goal of ensuring that "all people...will achieve their optimal lifespan with *the best possible quality of health* in every stage of life" (2). This emphasis on wellness reflects CDC's belief that the mere absence of disease is not synonymous with *the best possible quality of health.*

To help people achieve wellness throughout their lives, CDC is adopting a public health model that makes life stages the framework for its programs. Until recently, both public health and clinical medicine generally focused on specific diseases or conditions such as cancer, heart disease, disabilities, or birth defects. In the case of public health, this framework was established, in part, because of funding considerations: it was easier to convince decision makers of the need for resources to combat specific diseases than for resources to promote wellness through the various stages of life.

The health model of wellness through the life stages is sometimes described as a holistic approach to public health (3), an approach that aims to help people maintain good mental and physical health throughout their lives rather than focusing on specific diseases or conditions. Certain medical specialties such as pediatrics and gerontology already focus on the wellness of patients at specific life stages and can serve as models for public health efforts. Some ask whether this model can be effective in improving the public's health or in obtaining resources. To help answer this question, CDC has renewed its emphasis on external reviews of its programs to ensure a rigorous accounting of progress toward program goals based on explicit criteria for success. If evidence from these reviews suggests that programs based on this model are indeed more effective than more narrowly focused programs, it is likely that policy makers will take notice and embrace this new paradigm.

Within CDC, the Coordinating Center for Health Promotion (CoCHP) and its constituent organizations — the National Center on Birth Defects and Developmental Disabilities and the National Center for Chronic Disease Prevention and Health Promotion with its National Office of Public Health Genomics — have accepted a leadership role in developing and evaluating programs based on the wellness throughout the life stages model. Several articles in this issue of *Preventing Chronic Disease (PCD) *describe promising public health efforts based at least in part on this model and illustrate how public health interventions can foster wellness at various life stages.

For example, Whitehead and Leiker (4) describe how the use of a case management protocol helped lower blood lead levels among children. Though the percentage of children with blood lead levels at or above 10 µg/dL, a concentration associated with adverse neurologic outcomes (5,6), declined sharply from the mid 1960s through the mid 1990s (7) as the result of public health efforts to remove lead from paint, gasoline, and other sources, elevated blood lead levels remain a problem for some groups of U.S. children. Controlling for baseline differences in blood lead levels and demographic characteristics, they measured the decline in blood lead concentrations among young children with initial concentrations in the range of 10–19 µg/dL who followed a case management protocol. Their results highlight the utility of the protocol in efforts to further reduce blood lead levels among U.S. children.

In another article, Horn et al (8) report on the success of an emergency department-based program that uses motivational interviewing (a counseling method in which people are encouraged to explore and resolve ambivalent feelings as a means of changing problem behaviors) to help adolescent patients stop smoking. Their findings suggest that this program is flexible and may be used to complement other smoking cessation programs. The authors also discuss the overall strengths and limitations of using motivational intervention programs such as the one they described. As Horn et al note, teen smoking is a major public health problem in the United States. The most recent data from CoCHP's Youth Risk Behavior Survey show that 23% of high school students reported having smoked during the previous 30 days (9). These young people will be at increased risk for numerous smoking-related health problems throughout all remaining stages of their lives unless they can be induced to stop smoking.

Wagner et al (10) describe the effectiveness of adding telephone and in-person appointments as a means of increasing follow-up rates among low-income adult women whose initial cervical cancer screening results were positive. They also estimate the cost-effectiveness of the intervention and discuss how such an intervention might be implemented in different settings, including areas with differing economic conditions. Although cervical cancer remains the second most common cancer among women worldwide and a leading cause of cancer deaths, screening has led to a significant decline in deaths from cervical cancer (11). Screening, however, is effective only when accompanied by follow-up among women with positive screening results, and low-income women in the United States are at increased risk for cervical cancer in part because their screening follow-up rates are lower than those of the general population of U.S. women (12).

The extent to which all public health interventions, including those described in this issue of *PCD,* are actually implemented is a function of the allocation of limited public health resources and the prioritization of competing public health needs. One factor traditionally considered when making these calculations is the burden of disease and death caused by any given condition or disease. In this issue of *PCD*, Beverly Levine (13) discusses the use of the population-attributable fraction (PAF) metric to estimate the health burden associated with obesity (14). Levine elucidates the two common interpretations of PAF, clearly defines its legitimate use and meaning, and explains its limitations. Her discussion gives public health professionals and epidemiologists an opportunity to reevaluate and improve their approaches to prioritization in the context of known interventions.

In addition to contributing to this issue of *PCD*, CoCHP sponsored the 2006 National Health Promotion Conference, which highlighted the importance of the life-stages approach to wellness and health promotion. The theme of the conference, "Innovations in Health Promotion: New Avenues for Collaboration," also stressed CDC's desire to work with partners to leverage its investment in the public's health throughout all stages of life*.* Speakers included Jane Brody, author and *New York Times* columnist, who discussed how health promotion and wellness might be approached by encouraging Americans to engage in more healthful habits and practices. Kim Peek, who inspired the 1988 Oscar-winning movie *Rain Man,* and his father shared the challenges and opportunities of a person living with a disability. Bill Novelli, CEO of the American Association of Retired Persons, discussed wellness in the context of an aging population, including the challenges faced by aging people with disabilities. And Dr Edward Hill, past president of the American Medical Association, discussed ways to promote wellness in our population using important channels for communicating health messages, including physician visits, school health education, and adult information campaigns.

This issue of *PCD* and the National Health Promotion Conference in September both demonstrate a broad commitment throughout the public health community to promote health and wellness among people at all stages of life, a commitment that is also reflected in CDC's programs.
